# Supplementary material for: Context-dependent effects of relative temperature extremes on bill morphology in a songbird
Source: R Soc Open Sci. 2020 Apr 15;7(4):192203. doi: 10.1098/rsos.192203 (PMC7211890; doi:10.1098/rsos.192203)
Supplement: Additional methods and results [file rsos192203supp1.docx]

**Supplementary information for: Context-dependent effects of relative temperature extremes on bill morphology in a songbird**

Katie LaBarbera, Kyle J. Marsh, Kia R. R. Hayes, Talisin T. Hammond

**Obtaining environmental measures for each specimen**

We obtained records for four monthly climate variables from the PRISM historical climate dataset (PRISM climate group 2015): precipitation and mean, minimum, and maximum temperature. Temperature and precipitation are standard measures of abiotic climate (Danner and Greenberg 2015). The PRISM historical dataset provides GIS raster files containing the monthly means of the four climate variables as measured over 4 km-by-4 km grid cells across California. To determine which environmental conditions were associated with a given specimen, we used the latitude and longitude at which each specimen was collected to assign a raster cell. A buffer code was used to convert environmental values for each cell to those for a circle with a radius of 15 km centered on the collection locality for each specimen. We selected this area as a large enough distance to encompass dispersal movements and habitat within an individual juncos' perceptual range, including habitat to which the individual might move if the immediate area became inhospitable, while still small enough to represent the individual experience of these relatively sedentary birds (Chandler et al. 1994; Nolan et al. 2002). For each calendar month, values for cells entirely within this circle were averaged to generate a single mean value per variable; raster cells that were only partially located within the 15 km radius were not included in these analyses. This procedure was performed for each of the five years prior to the collection date of a specimen.

**Multicollinearity**

Collinearity and multicollinearity have the potential to confound the results of statistical models. We therefore avoided including variables that were highly correlated, and tested our model for multicollinearity using the variance inflation factor (VIF). Measures of the mean minimum and maximum temperatures over the previous year were strongly correlated with mean temperature over the previous five years, and substantially correlated with each other (Fig. S1); therefore, we did not include these in the model (also see "Unstandardized measures of maximum and minimum temperatures," below).

**Figure S1.** Correlations between climate variables. The variables "1yr_tmax" and "1yr_tmin" were excluded from statistical analysis due to correlation with each other and with "5yr_tmean".

High VIF values indicate that multicollinearity is likely to negatively influence a statistical model. Although there is some disagreement over what cutoff value should be used for VIFs, it is generably considered that VIFs >5 are of concern, and VIFs >10 are unacceptable (Tomaschek et al. 2018). All of the VIFs in our model were <4 (Table S1), indicating no major effects of multicollinearity in our analysis. Additionally, all VIFs >2 were for interaction terms and the individual factors involved in those interaction terms, which is expected (a variable is naturally somewhat correlated with an interaction that includes itself) and should not affect the results of the model (Allison 2012).

**Table S1.** Variance inflation factors for the variables included in our analysis.

| **Variable** | **VIF** |
| --- | --- |
| relative min temp | 2.69 |
| relative max temp | 3.22 |
| mean temp | 1.23 |
| relative min temp x mean temp | 1.35 |
| relative max temp x mean temp | 1.43 |
| temp std dev | 1.60 |
| relative min temp x recent precip | 2.36 |
| relative max temp x recent precip | 3.53 |
| recent precip | 1.55 |
| month | 1.19 |
| tarsus length | 1.07 |
| sex | 1.06 |
| subspecies | 1.84 |

**Unstandardized measures of maximum and minimum temperature**

Unstandardized measures of minimum and maximum temperature are of interest because heat loss through the bill depends on absolute, not relative, temperature. We tried running our statistical model with relative Tmin and relative Tmax replaced by the unstandardized mean Tmin and mean Tmax for the previous year: specifically, a generalized additive mixed model with bill surface area as the response, a nonlinear smooth term of latitude and longitude to control for spatial autocorrelation, year of collection as a random effect, and the following as fixed effects: mean temperature over five years; temperature standard deviation over five years; precipitation for the previous year; unstandardized mean maximum and minimum temperature for the previous year, and their interactions with mean temperature and with precipitation for the previous year; month of collection; subspecies; tarsus, to control for effects of overall body size; and sex. However, this model could not be run due to multicollinearity (Table S2).

**Table S2.** Variance inflation factors for the variables in a second analysis with different measures of minimum and maximum temperatures, which was not run due to high VIFs indicating issues of multicollinearity.

| **Variable** | **VIF** |
| --- | --- |
| min temp | 14.66 |
| max temp | 14.14 |
| mean temp | 31.43 |
| min temp x mean temp | 8.01 |
| max temp x mean temp | 8.27 |
| temp std dev | 3.15 |
| min temp x recent precip | 4.93 |
| max temp x recent precip | 4.28 |
| recent precip | 1.39 |
| month | 1.18 |
| tarsus length | 1.07 |
| sex | 1.06 |
| subspecies | 1.66 |

The removal of 5yr Tmean from the model reduced VIFs substantially, but two VIFs were still >5, rendering multicollinearity a concern. An ANOVA indicated that further removing both interaction terms from the model would not significantly reduce the model's explanatory power (χ^2^=1.33, df=2, *P*=0.514); and in that model, omitting Tmean *and* all interaction terms, VIFs were at acceptable levels (Table S3).

**Table S3.** Variance inflation factors for the variables in a third analysis with different measures of minimum and maximum temperatures, and with tmean and interactions omitted.

| **Variable** | **VIF** |
| --- | --- |
| min temp | 3.58 |
| max temp | 3.55 |
| temp std dev | 2.77 |
| recent precip | 1.24 |
| month | 1.15 |
| tarsus length | 1.06 |
| sex | 1.05 |
| subspecies | 1.59 |

This model found no relationship between climate variables and bill surface area (Table S4).

**Table S4.** An alternative model

| **Variable** | **Estimate±SE** | **t** | ***P*** |
| --- | --- | --- | --- |
| min temp | -0.161±0.180 | -0.89 | 0.372 |
| max temp | 0.060±0.141 | 0.42 | 0.673 |
| temp std dev | -0.024±0.517 | -0.05 | 0.964 |
| recent precip | -0.006±0.017 | -0.38 | 0.704 |
| month | -0.036±0.179 | -0.20 | 0.838 |
| subspecies (*J. h. thurberi*) | -3.500±1.040 | -3.36 | <0.001* |
| tarsus length | 1.669±0.450 | 3.71 | <0.001* |
| sex (male) | 1.533±0.501 | 3.06 | 0.002* |

**Analysis with wing chord instead of tarsus length as the proxy for body size**

Because tarsus length is necessarily an imperfect proxy for overall body size, and is potentially itself influenced by Allen's Rule, we re-ran our analysis with wing chord replacing tarsus length, to check whether our results are robust to the proxy for body size used.

This analysis yielded essentially the same results as the analysis with tarsus length, with significant effects of the interaction terms in the same direction as the other model, as well as effects of subspecies and sex. Where tarsus length was significantly related to bill surface area, however, wing chord was not. This may be because wing chord is less tightly correlated with body size than is tarsus length, or because both tarsus length and bill surface area are responding to climatic conditions as per Allen's Rule. Even in the latter case, the robustness of our results indicates that the patterns of response of bill surface area to climate are not solely driven by correlation with tarsus length.

**Table S5.** Bill surface area was influenced by temperature, precipitation, subspecies, and sex. Relative temperature minimum and maximum interacted with mean temperature and with precipitation. Asterisks indicate statistical significance. N = 516 individuals.

| **Variable** | **Estimate±SE** | **t** | ***P*** |
| --- | --- | --- | --- |
| relative min temp | -4.58±1.87 | -2.45 | 0.015* |
| relative max temp | 3.11±2.46 | 1.26 | 0.207 |
| mean temp | 0.06±0.11 | 0.55 | 0.581 |
| relative min temp x mean temp | 1.66±0.52 | 3.19 | 0.002* |
| relative max temp x mean temp | -1.46±0.56 | -2.62 | 0.009* |
| temp std dev | 0.23±0.42 | 0.56 | 0.575 |
| recent precip | -0.02±0.02 | -1.44 | 0.151 |
| relative min temp x recent precip | 0.19±0.06 | 3.03 | 0.003* |
| relative max temp x recent precip | -0.17±0.08 | -2.16 | 0.031* |
| month | -0.03±0.17 | -0.18 | 0.859 |
| subspecies (*J. h. thurberi*) | -3.51±1.03 | -3.40 | 0.007* |
| wing chord | 0.13±0.11 | 1.20 | 0.229 |
| sex (male) | 1.49±0.65 | 2.31 | 0.021* |

**Testing for Bergmann's Rule**

Bergmann's Rule states that animals are overall larger in size at lower temperatures to conserve heat (Danner and Greenberg 2015). If juncos conform to this by having larger body sizes at lower temperatures, that relationship in combination with allometrically-scaling bill size could contribute to the results we found that do not agree with Allen's Rule. We tested for this possibility with generalized additive mixed models of two proxies for overall body size: tarsus length and wing chord. Each of these models included five-year mean temperature, month, subspecies, and sex as fixed effects, year as a random effect, and the interaction between latitude and longitude as a smooth term to account for spatial autocorrelation.

#model of tarsus length

> b2 <- gamm4(tarsus ~ X5yr_tmean + X5yr_tsd + s(lat,long) + sex + thur + month, random = ~ (1|year), data = summ1)

**Table S6**. The model of tarsus length.

| **Variable** | **Estimate±SE** | **t** | ***P*** |
| --- | --- | --- | --- |
| mean temp | 0.01±0.01 | 0.26 | 0.793 |
| temp std dev | 0.03±0.04 | 0.83 | 0.409 |
| month | -0.02±0.02 | -1.40 | 0.161 |
| subspecies (*J. h. thurberi*) | -0.14±0.09 | -1.47 | 0.143 |
| sex (male) | 0.21±0.05 | 4.44 | <0.001* |

#model of wing chord

> b2 <- gamm4(wing ~ X5yr_tmean + X5yr_tsd + s(lat,long) + sex + thur + month, random = ~ (1|year), data = summ1)

**Table S7**. The model of wing chord.

| **Variable** | **Estimate±SE** | **t** | ***P*** |
| --- | --- | --- | --- |
| mean temp | 0.03±0.05 | 0.60 | 0.551 |
| temp std dev | 0.15±0.20 | 0.77 | 0.441 |
| month | 0.10±0.07 | 1.42 | 0.156 |
| subspecies (*J. h. thurberi*) | 1.56±0.46 | 3.42 | <0.001* |
| sex (male) | 3.84±0.20 | 19.04 | <0.001* |

Neither tarsus length nor wing chord is significantly associated with any of the tested climate variables (Tables S6-S7). Therefore, there is no evidence that Bergmann's Rule is acting on these birds or that it can explain the patterns of bill surface area response to temperature.

**REFERENCES**

P Allison. "When can you safely ignore multicollinearity?" Statistical Horizons (website), 2012. https://statisticalhorizons.com/multicollinearity. Accessed 21 November 2019.

CR Chandler, ED Ketterson, V Nolan, and C Ziegenfus, "Effects of testosterone on spatial activity in free- ranging male dark-eyed juncos, Junco hyemalis," *Animal Behaviour*, vol. 47, pp. 1445-1455, 1994.

RM Danner and R Greenberg, "A critical season approach to Allen’s rule: bill size declines with winter temperature in a cold temperate environment," *Journal of Biogeography*, vol. 42, no. 1, pp. 114-120, 2015.

V Nolan et al., "Dark-eyed junco (Junco hyemalis)," in *The birds of North America online*, A Poole, Ed. Ithaca: Cornell Lab of Ornithology, 2002.

F Tomaschek, P Hendrix, and RH Baayen. "Strategies for addressing collinearity in multivariate linguistic data." *Journal of Phonetics*, 71, 249-267, 2018.

PRISM Climate Group. (2015) [Online]. <http://prism.oregonstate.edu>
